# Correction: Gold nanoparticle coatings as efficient adenovirus carriers to non-infectable stem cells

**DOI:** 10.1039/c9ra90008j

**Published:** 2019-02-07

**Authors:** Yulan Hernandez, Rebeca Gonzalez-Pastor, Carolina Belmar-Lopez, Gracia Mendoza, Jesus M. de la Fuente, Pilar Martin-Duque

**Affiliations:** Departamento de Ciencias, Sección Química, Pontificia Universidad Católica del Peru PUCP Lima Peru; Instituto de Nanociencia de Aragón (INA), Universidad de Zaragoza 50018 Spain; Washington University in St Louis, School of Medicine St. Louis MO 63108 USA; Instituto Aragonés de Ciencias de la Salud 50009 Zaragoza Spain mpmartind.iacs@aragon.es; Instituto de Investigaciones Sanitarias de Aragón (IIS Aragón) 50009 Zaragoza Spain; Instituto de Ciencias de Materiales (ICMA), CSIC 50009 Zaragoza Spain; CIBER-BBN 28029 Madrid Spain; Fundación Araid 50001 Zaragoza Spain

## Abstract

Correction for ‘Gold nanoparticle coatings as efficient adenovirus carriers to non-infectable stem cells’ by Yulan Hernandez *et al., RSC Adv.*, 2019, **9**, 1327–1334.

The authors regret that some of the affiliation details were incorrect in the original article. The address provided in the footnote for the first author in the original article should actually be the first author’s current address (listed correctly here as affiliation a). The footnote for the second author in the original article should also have indicated that the address in the footnote is her current address (Washington University in St Louis, School of Medicine, St. Louis, MO 63108, USA).

The Royal Society of Chemistry apologises for these errors and any consequent inconvenience to authors and readers.

## Supplementary Material

